# Direct and indirect costs of cluster headache: a prospective analysis in a tertiary level headache centre

**DOI:** 10.1186/s10194-020-01115-4

**Published:** 2020-05-04

**Authors:** Andrea Negro, Paolo Sciattella, Valerio Spuntarelli, Paolo Martelletti, Francesco Saverio Mennini

**Affiliations:** 1 Department of Clinical and Molecular Medicine, Sapienza University, Rome, Italy; 2 Regional Referral Headache Centre, Sant’Andrea Hospital, Rome, Italy; 3 Economic Evaluation & HTA (CEIS- EEHTA), Faculty of Economics, University of Rome Tor Vergata, Rome, Italy; 4 Department of Statistical Sciences, Sapienza University of Rome, Rome, Italy; 5 Institute for Leadership & Management in Health, Kingston University London, London, UK

**Keywords:** Cost of illness, Cluster headache, Chronic cluster headache, Episodic cluster headache, Burden of disease, Resource utilization

## Abstract

**Background:**

Cluster headache (CH) is the most frequent trigemino-autonomic cephalgia. CH can manifest as episodic (ECH) or chronic cluster headache (CCH) causing significant burden of disease and requiring attack therapy and prophylactic treatment. The few data available on the economic burden of CH come from retrospective studies based on questionnaires, population surveys and medical insurance claims database. Although all these studies showed an important economic burden, they provided different estimates depending on variability of CH awareness and management, healthcare systems, available therapies and use of treatments according to different guidelines.

**Methods:**

This prospective study aimed to quantify the total direct and indirect cost of ECH and CCH over a cluster period, both for the patient and for the National Health System (NHS), using data from subjects who consecutively attended an Italian tertiary headache centre between January 1, 2018 and December 31, 2018.

**Results:**

A total 108 patients (89 ECH, 19 CCH) were included. Mean attack frequency was 2.3 ± 1.4 per day. Mean total cost of a CH bout was €4398 per patient and total cost of CCH was 5.4 times higher than ECH (€13,350 vs. €2487, *p* <  0.001). Direct costs represented the 72.1% of total cost and were covered for the 94.8% by the NHS. The costs for any item of expense were higher for CCH than for ECH (*p* <  0.001). Mean indirect costs for a CH bout were €1226 per patient and were higher for CCH compared to ECH (€3.538 vs. €732), but the difference was not significant. Days with reduced productive capacity impacted for the 64.6% of the total indirect costs. The analysis of the impact CH on work showed that 27%% of patients felt that CH had limited their career, 40% had changed their work pattern, 20% had changed their place of employment and 10% had lost a job due to the disease.

**Conclusion:**

Our results provide a valuable estimate of the direct and indirect costs of ECH and CCH in the specific setting of a tertiary headache centre and confirm the high economic impact of CH on both the NHS and patients.

## Introduction

Cluster headache (CH) is the most frequent of the so-called trigemino-autonomic cephalgias (TACs) and is the most severe of primary headaches. CH is characterized by excruciating unilateral pain lasting from 15 min to 3 h, with attacks occurring every other day up to eight times a day for weeks or months during active cluster periods, followed by periods of remission. Attacks are accompanied by a sense of restlessness or agitation and ipsilateral cranial autonomic features common to all TACs, such as conjunctival injection and/or lacrimation, nasal congestion and/or rhinorrhea, eyelid oedema, forehead and facial sweating, and miosis and/or ptosis [1]. The age of onset is typically between 20 and 40 years with a 3-fold male preponderance. CH is relatively rare compared with other primary headaches, as demonstrated by a meta-analysis of 15 studies in 10 countries that estimated a worldwide lifetime-prevalence of around 1 per 1000 in all age groups [2]. There are 2 types of CH: episodic CH (ECH) and chronic CH (CCH). According to the third edition of the International Classification of Headache Disorders (ICHD3) [1], patients with ECH suffer from headache attacks that occur in periods ranging from 7 days to 1 year, separated by pain-free periods lasting at least 3 months. CH occurs in 85–90% of patients as episodic with an average bouts duration of 8.6 weeks [3]. The remaining 10–15% of patients suffer from CCH), without such periods of remission.

Almost a third of the patients with ECH at onset develops CCH 10 years later and the same proportion of patients with CCH at onset turns into episodic within 10 years [4]. Unfortunately, half of patients with CCH at onset still have CCH after 20 years or more [4] and a considerable proportion often resist pharmaceutical treatments leading to refractory CCH as defined by the European Headache Federation (EHF) [5].

The pathogenesis of CH is incompletely understood, although evidence suggests that the trigeminovascular system and neurogenic inflammation play important roles and hypothalamic activation is suspected to be a key factor in the generation of attacks [6]. Treatment of CH is based exclusively on pharmacological measures and consists of two basic principles: symptomatic therapy taken at the time of an attack and preventive treatment to suppress further attacks. When a new cluster bout begins, both acute symptomatic and preventive therapy should be initiated. In addition to preventive medication, transitional therapies (e.g., oral steroids and suboccipital steroid injections) are often used to achieve short-term symptom improvement until the preventive drug dose can be increased and becomes effective. This therapeutic approach is particularly suitable for those patients with a high frequency of attacks.

CH has often been referred to as “suicide headache” due to the excruciating pain of the attacks and although these are periodic in most cases, the personal burden can be considerable due to lifestyle restrictions during the bouts, increased use of health care and the negative impact on work [7]. The few data available on the economic burden of CH come from retrospective studies based on questionnaires, population surveys and medical insurance claims database [8–10]. Although all these studies showed an important economic burden, they provided different estimates depending on variability of CH awareness and management, healthcare systems, available therapies and use of treatments according to different guidelines.

Therefore, this study was conducted in a tertiary headache centre to estimate the total cost (direct and indirect) of treating ECH and CCH over a cluster period and to determine the economic burden for patients and the National Health System (NHS).

## Methods

### Study design

The study is a prospective and cross-sectional evaluation of the direct and indirect costs of ECH and CCH, carried out at the outpatient Regional Referral Headache Centre, Department of Clinical and Molecular Medicine, Faculty of Medicine and Psychology, Sapienza University of Rome.

The study population included all patients with CH who consecutively attended our tertiary level headache center between January 1, 2018 and December 31, 2018. The headache diaries designed specifically for CH were given to participants to record the amount and duration of use (e.g., oxygen inhalation) of the prescribed acute, preventive and oral transitional treatments. Headache diaries were also used to record days off work and days with reduced work efficiency. Patients’ electronic medical records (EMRs) were used to collect further information, such as the number of visits to our headache centre, other specialist visits, diagnostic tests, therapeutic procedures performed at our clinic (e.g. steroid suboccipital injections) and admissions to the emergency department (ED). Patients’ EMRs are updated at each visit with information obtained from the patient and with data extracted from the headache diary.

Inclusion criteria were: 1) diagnosis of CH (according to ICHD3 [1]); 2) age ≥ 18 years at the time of enrollment; 3) continuous treatment for the entire duration of the cluster bout; and 4) complete compilation of the headache diary reporting medication consumptions.

### Data analysis

The data collected included demographic characteristics, medical history of CH, number of specialist visits, number of diagnostic tests (e.g. electrocardiogram and brain magnetic resonance), medication consumption (acute, transitional and preventive drugs) and number of ED admissions. In Italy, ED visits are fully funded by the NHS, while specialist visits, diagnostic tests, hospital therapeutic procedures and medications may be partially funded by the NHS or fully paid by the patient. The use of drugs has been quantified by analyzing patients’ headache diary records. Similarly, indirect costs due to days of absence from work and days with reduced work efficiency were assessed analyzing the data recorded by participants in their diaries.

### Economic analysis

The costs have been estimated from both societal and patient perspective. Intangible costs were not included in this evaluation. The costs have been calculated for the entire duration of a CH period. All costs are expressed in Euros and adjusted for the year 2019. Estimates of direct costs are the total of everything that has been paid or reimbursed by the NHS plus payments of own pocket money. Unit costs have been collected from publicly available sources in the calendar year 2019. The Regional Tariff Nomenclator for Outpatient Specialist Services was considered for outpatient specialist services [11] while each ED visit was considered to be €241 [12]. The costs of the drugs have been estimated using the reimbursement price of the Regional Health System for the classes of drugs charged to the NHS [13], while the costs of classes of drugs partially charged to the NHS or totally charged to the patient have been identified by a private site for health care professionals.

In addition, indirect costs caused by disability have been calculated according to the “cost of illness” methodology among gainfully employed subjects, both full-time and part-time [14]. The daily cost was calculated from the societal perspective using average labor costs. The cost of illness has been calculated by multiplying resource consumption and estimated prices.

### Statistics

The demographic and clinical characteristics, the number of specialist visits, the number of diagnostic tests, the consumption of drugs (reimbursed and not reimbursed by the NHS) and the number of ED visits have been assessed in a descriptive way. The categorical data were summarized by numbers and percentages, the continuous data by mean ± standard deviation (SD). The descriptive statistics are presented as means ± SD. The presence of statistically significant differences between ECH and CCH patients was evaluated by Chi-square test and Fisher exact test, when appropriate, for proportion, Student’s T tests for normal distributions and Mann-Whitney tests for non-normal distributions. All analyses were performed using the SAS statistical package, version 9.4 (SAS Institute Inc., Cary, NC, USA).

## Results

### Patients characteristics

The sample analyzed consisted of 108 patients, 9 (8.3%) women and 99 (91.7%) men, aged between 22 and 76 years. Patient characteristics are shown in Table 1. The average age of men was 44.1 ± 11.8 years and of women was 36.2 ± 12.3 years. The age at onset of CH was 30 ± 11 years. According to the ICHD-III diagnostic criteria, 89 (82.4%) subjects had ECH and 19 (17.6%) had CCH. On average, patients had 2.3 ± 1.4 attacks per day (CCH: 2.2 ± 1.7, ECH: 2.4 ± 1.3) with a range from 0.4 (3 per week) to 6 attacks per day. The mean duration of bouts in the total population was 14.8 ± 17.9 weeks with a minimum of 1 week to a maximum of 12 months. The mean duration of bouts among ECH patients was 6.8 ± 5.1 weeks with a minimum of 1 week to a maximum of 6 months.

**Table 1:** Patients characteristics: demographic, occupational and medical

Diagnosis	ECH (*n = 89*)	CCH (*n = 19*)	Total (*n = 108*)	***p-value***
Men, *n. (%)*	81 (91)	18 (95)	99 (92)	N.S.
Women, *n. (%)*	8 (9)	1 (5)	9 (8)	
Age, *years*	43.4 ± 12.5 (22–76)	43.8 ± 9.5 (23–62)	43.4 ± 12.0 (22–76)	N.S.
Level of education, *n. (%)*				
*Primary*	4 (4)	0 (0)	4 (4)	N.S.
*Secondary*	35 (39)	7 (37)	42 (39)	
*Degree*	50 (56)	12 (63)	62 (57)	
Gross annual income *(€)*				
*0*	21 (24)	7 (37)	28 (26)	N.S.
*< 10,000*	19 (21)	5 (26)	24 (22)	
*10,000 - 24,999*	32 (36)	3 (16)	35 (32)	
*≥ 25,000*	17 (19)	4 (21)	21 (19)	
Employment status, *n. (%)*				
*Employed (full-time)*	48 (53.9)	7 (36.8)	55 (50.9)	0.016
*Employed (part-time)*	20 (22.5)	5 (26.3)	25 (23.1)	
*Retired*	6 (6.7)	0 (0)	6 (5.6)	
*Unemployed*	7 (7.9)	7 (36.8)	14 (13)	
*Housewife/student*	8 (9)	0 (0)	8 (7.4)	
Age at onset, *years*	30.5 ± 11.4 (14–64)	29.9 ± 10.2 (18–56)	30.4 ± 11.2 (14–64)	N.S.
Duration of disease, *years*	12.8 ± 10.0 (0–37)	13.9 ± 7.1 (0–30)	13,0 ± 9.5 (0–37)	N.S.
Diagnostic delay, *years*	4.2 ± 3.6 (0–16)	2.7 ± 1.8 (0–7)	3.9 ± 3.4 (0–16)	N.S.
New CH diagnosis, *n. (%)*	30 (33.7)	1 (5.3)	31 (28.7)	0.012
Frequency of attacks, *per day*	2.4 ± 1.3 (0.4–5.0)	2.2 ± 1.7 (0.4–6.0)	2.3 ± 1.4 (0.4–6.0)	N.S.
Duration of attack without treatment, *min*.	64.3 ± 32.2 (20–150)	79.7 ± 31.3 (35–150)	67.0 ± 32.4 (20–150)	0.30
Duration of attack with treatment, *min*.	13.7 ± 5.6 (5–40)	13.5 ± 4.7 (8–25)	13.6 ± 5.4 (5–40)	N.S.
Duration of bouts, *weeks*	6.8 ± 5.1 (1–25)	52.0 ± 0 (52)	14.8 ± 17.9 (1–52)	< 0.0001
Laterality, left, *n. (%)*	46 (52)	8 (42)	54 (50	N.S.
CH symptoms, *n. (%)*				
*conjunctival injection and/or lacrimation*	45 (50.6)	12 (63.2)	57 (52.8)	N.S.
*nasal congestion and/or rhinorrhoea*	54 (60.7)	8 (42.1)	62 (57.4)	N.S.
*eyelid oedema*	66 (74.2)	15 (78.9)	81 (75)	N.S.
*forehead and facial sweating*	41 (46.1)	8 (42.1)	49 (45.4)	N.S.
*miosis and/or ptosis*	40 (44.9)	11 (57.9)	51 (47.2)	N.S.
*sense of restlessness or agitation*	65 (73)	14 (73.7)	79 (73.1)	N.S.

*CCH* Chronic cluster headache, *ECH* Episodic cluster headache

### Specialist visits

Patients enrolled in the study consulted our outpatient headache centre with an average number of 3.0 ± 1.9 visits (range 1–9) during the CH period (Table 2). The average number of headache visits was higher for CCH (6.7 ± 1.5; range: 4–9) than ECH (2.2 ± 0.7, range 1–5; *p* <  0.0001). A cardiologist was consulted by 13 (68.4%) CCH patients and by 23 (11.2%) ECH patients (*p* <  0.0001).

**Table 2:** Healthcare resource use

Diagnosis	ECH (*n = 89*)	CCH (*n = 19*)	Total (*n = 108*)	***p-value***
Headache centre visits, *n.*	2.2 ± 0.7 (1–5)	6.7 ± 1.5 (4–9)	3.0 ± 1.9 (1–9)	< 0.0001
Cardiology visits, *n. (%) patients*	10 (11.2)	13 (68.4)	23 (21.3)	< 0.0001
ED visits, *n. (%) patients*	14 (15.7)	11 (57.9)	25 (23.1)	< 0.0001
ED visits, *n. per patient*	0.2 ± 0.6 (0–3)	0.7 ± 0.7 (0–2)	0.3 ± 0.6 (0–3)	0.000
ECG, *n. (*%) patients	53 (59.6)	18 (94.7)	71 (65.7)	0.002
ECG, *n. per patient*	0.6 ± 0.7 (0–3)	2.5 ± 1.2 (0–4)	0.9 ± 1.1 (0–4)	< 0.0001
Brain MRI, *n. (%) patients*	21 (23.6)	16 (84.2)	37 (34.3)	< 0.0001
Brain MRI, *n. per patient*	0.2 ± 0.4 (0–1)	0.8 ± 0.4 (0–1)	0.3 ± 0.5 (0–1)	< 0.0001
Acute medications, *n. (%)*				
*Sumatriptan 6 mg*	56 (62.9)	17 (89.5)	73 (67.6)	0.030
*Zolmitriptan 5 mg*	19 (21.3)	1 (5.3)	20 (18.5)	N.S.
*Oxygen*	71 (79.8)	19 (100)	90 (83.3)	0.038
*Combinations*	57 (64)	18 (94.7)	75 (69.4)	0.007
Transitional treatments, *n. (%)*				
Oral corticosteroids	29 (32.6)	0 (0)	29 (26.9)	0.002
G.O.N. block	8 (9)	15 (78.9)	23 (21.3)	< 0.0001
Preventive medications, *n. (%)*				
*Verapamil*	62 (69.7)	15 (78.9)	77 (71.3)	N.S.
*Topiramate*	30 (33.7)	11 (57.9)	41 (38)	0.048
*Combinations*	51 (57.3)	18 (94.7)	69 (63.9)	0.001
Nutraceutics, *n. (%)*				
*Melatonin*	31 (34.8)	17 (89.5)	48 (44.4)	< 0.0001

*CCH* Chronic cluster headache, *ECH* Episodic cluster headache, *ECG* Electrocardiogram, *ED* Emergency department, *GON* Great occipital nerve, *MRI* Magnetic resonance imaging

The mean cost of specialist visits was €169 ± €114 (range: €53–527) per patient, covered by NHS for the 39%, and was significantly higher for CCH (€391 ± €77) than ECH (€122 ± €42; p <  0.0001) (Table 3).

**Table 3:** Direct costs per CH bout

Diagnosis	EEC (*n = 89*)		CCH (*n = 19*)		Total (*n = 108*)		***p-value***
	NHS	PRI	NHS	PRI	NHS	PRI	
Specialist visits	€48 ± €16 (21–103)	€74 ± €25 (32–160)	€153 ± €30 (103–207)	€237 ± €47 (160–320)	€66 ± €45 (21–207)	€103 ± €69 (32–320)	< 0.0001
ED visit	€51 ± €133 (0–723)	–	€165 ± €162 (0–482)	–	€71 ± €145 (0–723)	–	0.000
Diagnostic tests	€113 ± €161 (0–506)	€24 ± €27 (0–111)	€426 ± €156 (0–558)	€94 ± €34 (0–133)	€168 ± €200 (0–558)	€36 ± €39 (0–133)	< 0.0001
Acute medications	€1387 ± €1938 (14–8724)	–	€8314 ± €6822 (362–25,024)	–	€2606 ± €4236 (14–25,024)	–	< 0.0001
Transitional treatments	€7 ± €15 (0–60)	€3 ± €9 (0–32)	€41 ± €22 (0–52)	€25 ± €13 (0–32)	€13 ± €21 (0–60)	€7 ± €13 (0–32)	< 0.0001
Preventive medications	€40 ± €58 (0–371)	€8 ± €15 (0–60)	€275 ± €85 (153–445)	€82 ± €29 (0–91)	€81 ± €110 (0–445)	€21 ± €33 (0–91)	< 0.0001
**Total**	€1647 ± €2064 (34–9629)	€109 ± €60 (32–299)	€9374 ± €6898 (1382-26,147)	€438 ± €71 (315–554)	€3006 ± €4500 (34–26,147)	€166 ± €140 (32–554)	< 0.0001
	**€1755 ± €2110** **(66–9927)**		**€9812 ± €6932** **(1755-26,626)**		**€3173 ± €4609** **(66–26,626)**		**< 0.0001**

*CCH* Chronic cluster headache, *ECH* Episodic cluster headache, *ED* Emergency department, *NHS* National health system, *PRI* Private cost

### Diagnostic tests

The use of at least one diagnostic during the bout involved 71 (65.7%) patients (Table 2). Seventy-one (65.7%) patients received at least one electrocardiogram (ECG). Higher proportion of CCH patients had prescribed an ECG compared to ECH patients (94.7.% vs. 59.6%; p 0.002). The mean number of ECG per patient was higher for CCH (2.5 ± 1.2) than ECH (0.6 ± 0.7; *p* <  0.0001). Thirty-seven (34.3%) patients underwent to brain magnetic resonance imaging (MRI). Higher proportion of CCH patients received a prescription for a brain MRI compared to ECH patients (84.2% vs. 23.6%; *p* <  0.0001). The mean number of brain MRI per patient was higher for CCH (0.8 ± 0.4) than ECH (0.2 ± 0.4; *p* <  0.0001).

The diagnostic tests had an average cost per patient of €204 ± €237 (range: €0–691), covered by the NHS for 82.4% (€168 ± €200). The cost was significantly higher for CCH (€520 ± € 188) than ECH (€137 ± €187; *p* <  0.0001) (Table 3).

### ED visits

During a bout, 25 (23.1%) of 108 patients entered the ED because of CH attack (Table 2). Higher proportion of CCH patients visited the ED compared to ECH patients (57.9% vs. 15.7%; *p* <  0.0001). The average number of ED visits per patient was higher for CCH (0.7 ± 0.7, range 0–2) than ECH (0.2 ± 0.6; range: 0–3; p <  0.0001).

The mean annual cost for the NHS relating to ED visits was €71 ± €145 (range: €0–723) per patient and the cost was higher for CCH (€165 ± €162) than ECH (€51 ± €133; p <  0.0001) (Table 3).

### Medications consumption

The allocation of intake of attack-aborting treatment and drugs is shown in Table 2. Regarding acute medications, CCH patients used significantly more sumatriptan 6 mg and oxygen than EEC patients (*p* = 0.030 and *p* = 0.038, respectively), while the use of zolmitriptan 5 mg did not differ between the two groups. Higher proportion of CCH patients used more than one medication to treat the headache (*p* = 0.007). Regarding preventive medications, CCH patients used significantly more topiramate than ECH patients (*p* = 0.048), while the use of verapamil did not differ between the two groups. Higher proportion of CCH patients used a combination of two drugs as prevention (*p* = 0.001). Similarly, the use of transitional treatments, both oral corticosteroids and steroid suboccipital injections, was significantly higher for CCH than ECH patients (*p* = 0.002 and *p* <  0.0001, respectively). CCH patients also used more melatonin than ECH patients (p <  0.0001).

The mean cost of acute medications for CH bout was €2606 ± €4236 (range: €14–25,024) per patient and was significantly higher for CCH (€8314 ± €6822) than ECH (€1387 ± €1938; *p* < 0.0001) (Table 3). The mean cost of preventive medications for CH bout was €102 ± €138 (range: €0–537) per patient and was significantly higher for CCH (€357 ± €74) than ECH (€48 ± €70; p < 0.0001). The mean cost of transitional treatments for CH bout was €20 ± €34 (range: €0–92) per patient and was significantly higher for CCH (€66 ± €35) than ECH (€10 ± €24; *p* < 0.0001).

### Impact on work

**Fig. 1:**
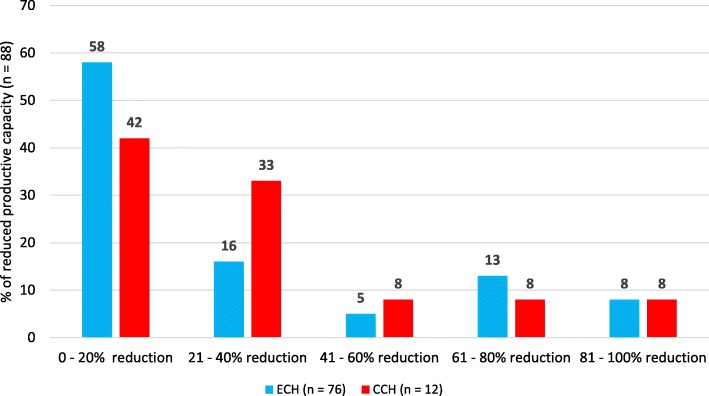
Reported work efficiency during a cluster period

**Fig. 2:**
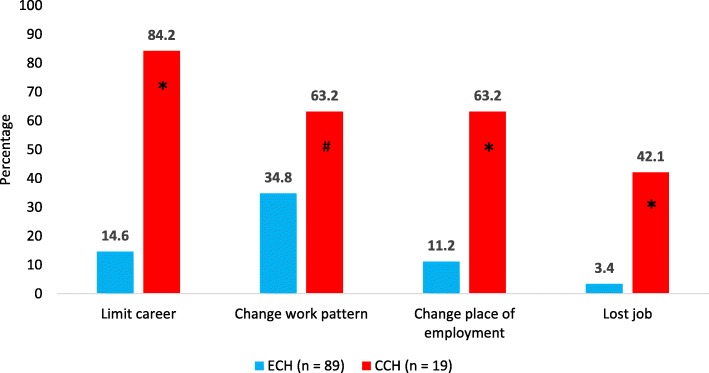
Reports of work changes due to cluster headache. Legend: * *p* < 0.0001; ^#^*p* = 0.022

The mean number of days off work within the bout due to CH was nearly 7 days. Statistically the number of absence days due to headache was significantly larger among CCH patients (15.2 ± 11.8) than ECH patients (5.6 ± 7.9; *p* < .0001) (Table 4). On the contrary, the proportion of days off work respect to the bout duration was higher for ECH patients than CCH patients (10.3% vs. 4.2%, respectively; *p* < .0001), due to the longer duration of CCH bout. Overall, CH was responsible of nearly 16 days of work with reduced capacity. The impact of CCH was higher than that of ECH (46.3 ± 72.2 vs. 10.8 ± 14.0 days, respectively), but the difference was not significant. The reduction of productive capacity at work is shown in Fig. 1. The reports of work changes due to CH are shown in Fig. 2.

**Table 4:** Impact on work and indirect costs

Diagnosis	ECH (*n = 77*)	CCH (*n = 12*)	Total (*n = 89*)	***p-value***
Days off work (or study), *n.*	5.6 ± 7.9 (1–45)	15.2 ± 11.8 (5–45)	6.9 ± 9.1 (1–45)	< 0.0001
Days off work (or study) / bout duration, *%*	10.3 ± 6.4 (2–31)	4.2 ± 3.2 (1–12)	9.5 ± 6.4 (1–31)	0.000
Days with reduced productive capacity, *n.*	10.8 ± 14.0 (0–70)	46.3 ± 72.2 (0–220)	15.6 ± 31.3 (0–220)	N.S.
Days with reduced productive capacity / bout duration, *%*	18.1 ± 16.3 (0–52)	12.7 ± 19.8 (0–60)	17.4 ± 16.8 (0–60)	N.S.
Days off work *(per bout)*	€339 ± €680 (0–3678)	€879 ± €1694 (0–5603)	€434 ± €952 (0–5603)	N.S.
Days with reduced productive capacity *(per bout)*	€393 ± €1352 (0–11,648)	€2659 ± €7818 (0–29,501)	€792 ± €3541 (0–29,501)	N.S.
**Total*(per bout)***	**€732 ± €1.928** **(0–15,326)**	**€3.538 ± €9420** **(0–34,865)**	**€1226 ± €4374** **(0–34,865)**	**N.S.**

*CCH* Chronic cluster headache, *ECH* Episodic cluster headache

The mean cost of absences from work for CH bout was €434 ± €952 (range: €0–5603) per patient and was higher for CCH (€879 ± €1694) then ECH (€339 ± €680), but the difference was not significant (Table 4). The mean cost attributed to days with reduced productive capacity for CH bout was €792 ± €3541 (range: €0–29,501) per patient and was higher for CCH (€2659 ± €7818) then ECH (€393 ± €1352), but the difference was not significant.

### Direct costs

**Fig. 3:**
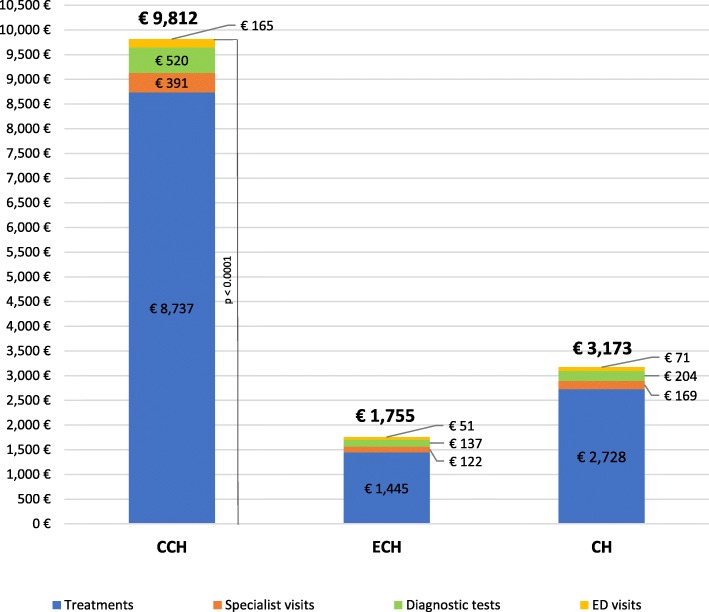
Direct costs per cluster headache patient

The mean direct costs of a CH bout were €3173 ± €4609 (range: €66–26,626) per patient and was covered for the 94.8% (€3006) by the NHS (Table 3). The main item of expenditure was represented by treatments that accounted for 86% (€2728), followed by diagnostic tests for 6% (€204), specialist visits for 5% (€169) and ED visits for 3% (€71) (Fig. 3). The mean direct costs of a CH bout were significantly higher for CCH (€9812 ± €6932) than ECH (€1755 ± €2110; *p* < 0.0001). There were small differences between the impact of the different expenditure items between CCH and ECH: 89% vs. 82% for treatments, 5% vs. 8% for diagnostic tests, 4% vs. 7% for specialist visits, and 2% vs. 3% for ED visits, respectively.

### Indirect costs

The mean indirect costs for a CH bout were €1226 ± €4374 (€0–34,865) per patient and were higher for CCH (€3.538 ± €9420) than ECH (€732 ± €1928), but the difference was not significant (Table 4). Days with reduced production capacity impacted for the 64.6% (€792) of the total indirect costs.

### Analysis of total costs

**Fig. 4:**
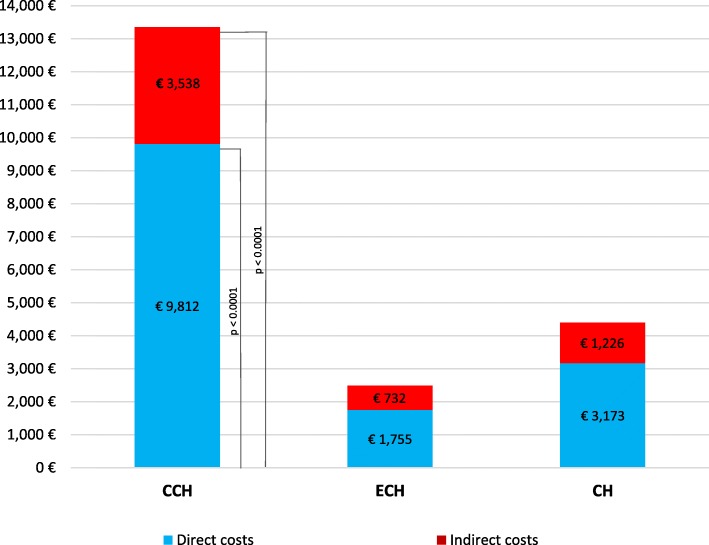
Total costs per cluster headache patient

The mean total costs (direct + indirect) for a CH bout were €4398 ± €7724 (66–51,281) per patient and the direct costs accounted for 72.1% (€3173) (Fig. 4). The total cost of CCH (€13,350 ± €13,991; range 2157-51,281) was 5.4 times higher than ECH (€2487 ± €3394; range 66–20,697; *p* < 0.001) and the difference in the total average costs of a CH bout between CCH and ECH was €10,863. The impact of direct costs on total expenditure was higher for CCH than ECH (73.5% vs. 70.6%), but the difference was not significant.

## Discussion

Our study provides a detailed quantification of the mean direct and indirect costs associated with ECH and CCH (assessed with the ICHD-3 [1]) in a large population of patients attending an Italian tertiary level headache centre. Direct costs accounted for nearly three fourth of the total costs and 94.8% (€3006) was funded by the NHS while patients had an annual personal expenditure of €167 as a contribution for specialist visits, diagnostic tests, and medications. The total cost of CCH was 5.4 times higher than that of ECH. The cost charged to the NHS was €9374 for CCH and €1647 for ECH, while the cost for the patient was €438 for CCH and €109 for ECH. The costs for any item of expense were higher for CCH than for ECH. In fact, compared to ECH patients, CCH patients made 3-times more specialist visits and ED visits and 4-times more ECG and brain MRI. Overall, these data indicate that CCH patients have an increased need for follow-up due to the higher severity of the condition and the higher dose of preventive medications.

Only one other European study has recently assessed the cost of CH in our same setting of a headache centre. In 2010, *Gaul* et al. [8] retrospectively assessed the direct and indirect costs of CH diagnosed according to the second edition of the ICHD [15] in a sample of 179 patients (72 CCH, 107 CCH) attending a German headache centre. Unfortunately, important differences in the methodology and in the voices of expenditure considered between their and our study may limit a proper comparison of the results. The authors assessed the cost of a 6-month period while we prospectively evaluated the costs related to the duration of a CH bout. Moreover, *Gaul* et al. included the costs related to some drugs (e.g. lithium, gabapentin, valproate), specialist visits (e.g. pain specialist, osteopath, neurosurgeon) and procedures (e.g. physiotherapy, massage and manual therapy, acupuncture, occipital nerve stimulation) that we did not use in our patients [8]. In their study, hospitalization for headache treatment (€24,086), rehabilitation (€18,556) and the costs of surgery and hospitalization for occipital nerve stimulation (€40,578) had an important impact on costs estimates. They estimated the total costs of a 6-month period in €10,985 per patient with CCH and €2583 per patient with ECH.

As regard to indirect costs, in our study they represented 27.9% of the total cost of CH bout and, as expected, they were higher for CCH than ECH (€3.538 vs. €732), although not significantly. Days with reduced production capacity impacted for 64.6% of total indirect costs. CCH patients made significantly more absences from work than ECH patients (15.2 ± 11.8 vs. 5.6 ± 7.9) and, although the cost of days off work was higher for CCH then ECH (€879 vs. €339), the difference was not significant. A previous analysis conducted by *Jensen* et al. on 85 CH patients (20% with CCH) in a Danish headache centre showed an important impact on work [9]. In detail, 39% of patients felt that CH had limited their career, 27% had changed their work pattern, 15% had changed their place of employment and 16% had lost a job due to the disease. In our study, we investigated the same aspects and found similar results (27%, 40%, 20% and 10%, respectively). In addition, we also analyzed the impact of the two forms of CH separately, showing a significantly higher burden in association with CCH. In detail, the ratio of the percentages of CCH and ECH patients for limitation of their career was 5.7:1, for the change of work pattern was 1.8:1, for the change of place of employment was 5.6:1 and for job loss was 12.4:1. When asked to quantify their work efficiency, 58% of ECH patients and 42% of CCH patients felt no or very few restrictions in their work efficiency, either because they had very effective and reliable treatment for the headaches or because most attacks occurred during night time and did not affect their daytime condition. Unexpectedly, we have not observed any differences in the severe reduction in production capacity between CCH and ECH.

## Methodological considerations

There are several important limitations that need to be considered when interpreting these results. Our study was conducted on a sample of patients attending a tertiary headache centre and our results may therefore not be representative of CH patients in the general population as specialist clinics usually see most disabled patients with a history of treatment failures and treatment attempts by general practitioners. However, most previous studies of CH have been conducted on patients from headache clinics. In addition, the proportion between ECH and CCH (82.4% and 17.6%, respectively) and duration of the disease (13 years) were similar to previous epidemiological series and our results can therefore be comparable to both studies conducted in the same setting of a headache clinic [9] and most other population-based studies [16, 17]. The higher proportion of CCH patients in a tertiary headache centre compared than in the general population may result in more people refractory to conventional treatment. In our population, 15 of the 19 CCH patients had refractory CCH according to the definition proposed by EHF [5]. Refractory patients use more healthcare resources and more acute treatments, and because of their higher and permanent disability compared to other patients, they show a greater work impairment leading to increased indirect costs. Unfortunately, the detailed analysis of this subgroup of patients has not been made. However, we believe that demographic and clinical CH characteristics of our patients’ sample are those typical of a cohort of CH and, therefore, our population may be comparable to other clinical populations [8, 17].

With regard to the cost analysis, we have calculated only the direct and indirect costs closely related to CH, but not the cost of conditions that are comorbid or secondary to CH or to its treatment, which can have a strong economic impact on the management of CH. Taking those aspects into account, the cost of headache would be even higher. Our economic analyses have been designed to capture only the costs associated with a CH bout, from the day of the onset until the end of the active period with suspension of the pharmacological treatment and final follow-up at our headache centre. Consequently, we have not assessed the cost of the referring general practitioners, which in Italy is entirely covered by the NHS. Likewise, we have not assessed the costs of physicians, resident doctors and nurses (fully covered by the NHS) or the costs of medications (e.g. lithium) and procedures (e.g. surgery, transcutaneous nerve stimulators and acupuncture) which for various reasons are not routinely used in our clinic. A further limitation of our economic analysis is that we have not calculated the indirect costs due to the impact of CH on family life.

An advantage of this study is the high validity of the headache diagnosis, which was assessed by a headache expert. In contrast, studies in which patients were identified by administrative claims as opposed to medical records showed the potential for misclassification of migraine or chronic migraine as CH [10]. In addition, administrative claims data may be subject to data encoding limitations and data entry error and, in some countries, may be limited only to individuals with employee health insurance or supplemental insurance, so that they cannot be representative of all CH patients.

To our knowledge, this study is the first to establish the diagnosis according to the most recent and currently used third version of the ICHD criteria, which presents important differences form the second edition used in previous studies [15].

However, the main advantage of this study is the adoption of a prospective design that has never before been used before for “cost of illness” studies for CH. In fact, recall bias is usually a major problem in retrospective clinical studies, even for highly debilitating diseases such as CH, for which one might expect most patients to be able to remember their attacks accurately. However, recall specific data on drug consumption and days off work or with reduced productivity is usually more unreliable. Instead, we have carried out a detailed analysis by collecting information on every single attack during the CH period and calculating in detail the changes in drug use. This method provides more reliable data on health resource utilization than other studies that have calculated the number of attacks by multiplying the frequency of attacks per day by the duration of the CH bout [8].

## Conclusions

Our results provide a valuable estimate of the direct and indirect costs of patients with ECH and CCH in the specific setting of a tertiary level headache centre and confirm the high economic impact of CH on both the NHS and patients. CCH Patients had more visits, diagnostic tests and drug use than patients with ECH, which led to a total cost of 5.4 times that of ECH.

Cost of illness studies become obsolete due to changing healthcare systems and new treatments become available. Governments and decision-makers should strongly support these investigations to reveal the true economic and social impact of this devastating pain disease, particularly when it is chronic.

## Data Availability

Dataset available from the corresponding author on reasonable request.

## Abbreviations

CH: Cluster headache; CCH: chronic cluster headache
DRG: Diagnosis related Group
ECG: Electrocardiogram
EHF: European Headache Federation
EMR: Electronic medical records
ECH: Episodic cluster headache
ED: Emergency department
GON: Great occipital nerve
ICHD: International Classification of Headache Disorders
MRI: Magnetic resonance imaging
NHS: National Health System
